# Beyond healthcare access: social deprivation and COVID-19 outcomes in dialysis patients in the provence-alpes-côte d’Azur region, France

**DOI:** 10.1186/s12879-026-12774-0

**Published:** 2026-02-10

**Authors:** Franck Mazoué, Sébastien Cortaredona, Adeline Crémades, Ghizlane Izaaryene, Bénédicte Devictor, Philippe Brunet, Stéphanie Gentile

**Affiliations:** 1 Cellule d’appui épidémiologique, registre REIN Provence-Alpes Côtes d’Azur, Hôpital de La Conception, Assistance Publique - Hôpitaux de Marseille, Marseille, France; 2 Aix-Marseille University, Public Health , Chronic Diseases and Quality of Life research Unit, 27 Boulevard Jean Moulin, 13385,cedex 5 EA 3279 Marseille, France; 3 Aix-Marseille Univ, IRD, SSA, MINES, Marseille, France; 4 Aix-Marseille Univ, SSA RITMES, Marseille, France; 5 IHU-Méditerranée Infection, Marseille, France; 6 Department of Nephrology and Renal Transplantation, Hôpital de La Conception, Assistance Publique - Hôpitaux de Marseille, Marseille, France; 7 Service d’Evaluation Médicale, Hôpital de La Conception, Assistance Publique - Hôpitaux de Marseille, Marseille, France

**Keywords:** COVID-19, Dialysis, FDep, Socioeconomic deprivation, ESRD

## Abstract

**Background:**

Socioeconomic deprivation has been consistently associated with worse COVID-19 outcomes, yet it remains unclear whether social gradients persist in populations receiving regular, highly structured, life-sustaining care. Dialysis patients provide a specific context to explore whether structural social determinants continue to shape epidemic vulnerability beyond healthcare access alone.

**Objective:**

To assess the association between socioeconomic deprivation and both COVID-19 infection and clinical severity among dialysis patients in the Provence-Alpes-Côte d’Azur (PACA) region during the pre-vaccination period (2020).

**Methods:**

We conducted a retrospective cohort study using the REIN registry including adult dialysis patients living in PACA in 2020. Area-level deprivation was measured using the French Deprivation Index (FDep) at the IRIS level. We analysed factors associated with (i) COVID-19 infection and (ii) severe COVID-19 among infected patients using multivariable models accounting for individual characteristics, comorbidities, dialysis modality, and contextual variables. A sensitivity analysis was performed by epidemic wave to assess robustness.

**Results:**

Higher socioeconomic deprivation was associated with increased risk of COVID-19 infection and with more severe clinical forms among infected patients, after adjustment for individual and contextual covariates. Associations were consistent across epidemic waves in sensitivity analyses, supporting the robustness of the findings.

**Conclusion:**

Social gradients in COVID-19 infection and severity persisted in a population benefiting from regular, continuous dialysis care, suggesting that structural social determinants (e.g. living conditions and deprivation-related vulnerabilities) play a critical role in epidemic risk. Beyond the COVID-19 pandemic, these findings provide lessons for epidemic preparedness and the management of socially vulnerable populations with chronic diseases, supporting the integration of deprivation indicators into routine care and epidemic preparedness strategies.

**Supplementary information:**

The online version contains supplementary material available at 10.1186/s12879-026-12774-0.

## Background

The World Health Organization (WHO) declared COVID-19 a global pandemic on 11 March 2020 [1]. As of September 2023, over 770 million confirmed cases and nearly 7 million deaths had been reported worldwide [2]. France recorded its first case in January 2020 and, by March 2021, more than 400,000 hospitalizations and nearly 70,000 in-hospital deaths, with a hospital mortality rate of 17% [3].

The country experienced multiple epidemic waves associated with different SARS-CoV-2 variants. The first two waves occurred between March and June 2020 (Wuhan-Hu-1 variant), and between September and December 2020 (B.1.160 variant) [3]. Nationwide vaccination campaigns began in January 2021, and by December of that year, 76.8% of the population had received at least two vaccine doses [3].

Early in the pandemic, several comorbidities were identified as risk factors for severe COVID-19, including diabetes, cardiovascular disease, hypertension, chronic kidney disease (CKD), and cancer. Older age and male sex were also associated with higher mortality [4–14].

Numerous studies have since highlighted that COVID-19 outcomes were not only shaped by clinical vulnerability but also by socioeconomic factors [15, 16]. Deprivation increased the likelihood of exposure to the virus, particularly through crowded housing, public transport reliance, and essential occupations. In addition, people living in poverty tend to have higher rates of chronic illness, reduced access to preventive care, and delayed health-seeking behaviors. These factors combine to increase the risk of both infection and severe outcomes.

However, most of this literature implicitly assumes that unequal access to care is a major driver of poor outcomes among socially deprived groups. In this regard, the case of dialysis patients in France presents a unique situation. These patients receive structured, regular (three times a week), and care covered by the national health insurance system, regardless of socioeconomic status. Once enrolled in the dialysis care pathway, patients benefit from regular and highly structured medical follow-up, delivered according to standardized national clinical protocols. In France, access to dialysis is organized within regional healthcare planning frameworks designed to ensure equitable territorial coverage. Once patients are enrolled in the dialysis care pathway, treatment delivery relies on standardized clinical protocols, regular in-person sessions, and quality indicators monitored at the national level [17, 18]. Unlike many other care pathways, dialysis treatment could not be interrupted during lockdown periods, as it is a life-sustaining therapy. Dialysis units therefore remained operational throughout the pandemic, ensuring continuity of care for patients, although under adapted organizational conditions.

This context provides a rare opportunity to test whether social inequalities continue to affect infection risk and severity when access to care is no longer a limiting factor. If socioeconomic deprivation still produces worse outcomes under these conditions, it would suggest the presence of deeper, structural determinants that act independently of healthcare access.

The French Renal Epidemiology and Information Network (REIN) registry has been monitoring dialysis patients nationwide since 2002, and initiated specific COVID-19 surveillance at the outset of the pandemic, with the aim of studying the incidence, lethality, and risk of death in this highly vulnerable population [19]. Dialysis patients are mostly elderly (mean age 71.0 years in 2021) and present multiple comorbidities, with nearly 60% having at least one cardiovascular condition; in addition, they must attend dialysis centers three times a week for treatment, which increases their potential exposure to infection [20]. Data collected through this surveillance revealed regional variations in COVID-19 prevalence and outcomes among dialysis patients, with higher incidence in some parts of the country—including the Provence-Alpes-Côte d’Azur (PACA) region—regardless of age.

In this study, we aimed to examine whether socioeconomic deprivation at the area level influenced both the prevalence and severity of COVID-19 among dialysis patients in the PACA region during the first year of the pandemic, before vaccines became widely available. Our objective was to test whether social gradients persist in a population with regular and standardized follow-up once enrolled in dialysis, thereby contributing to a broader understanding of how structural determinants shape vulnerability to infectious disease.

## Materials and methods

### Study design, setting, and population

This retrospective study was conducted of all adult dialysis patients over 18 years old treated in the PACA region between 1 March and 31 December 2020. This time period (first year of the pandemic before vaccination) was chosen to avoid any vaccination-related confounding bias.

**Fig. 1:**
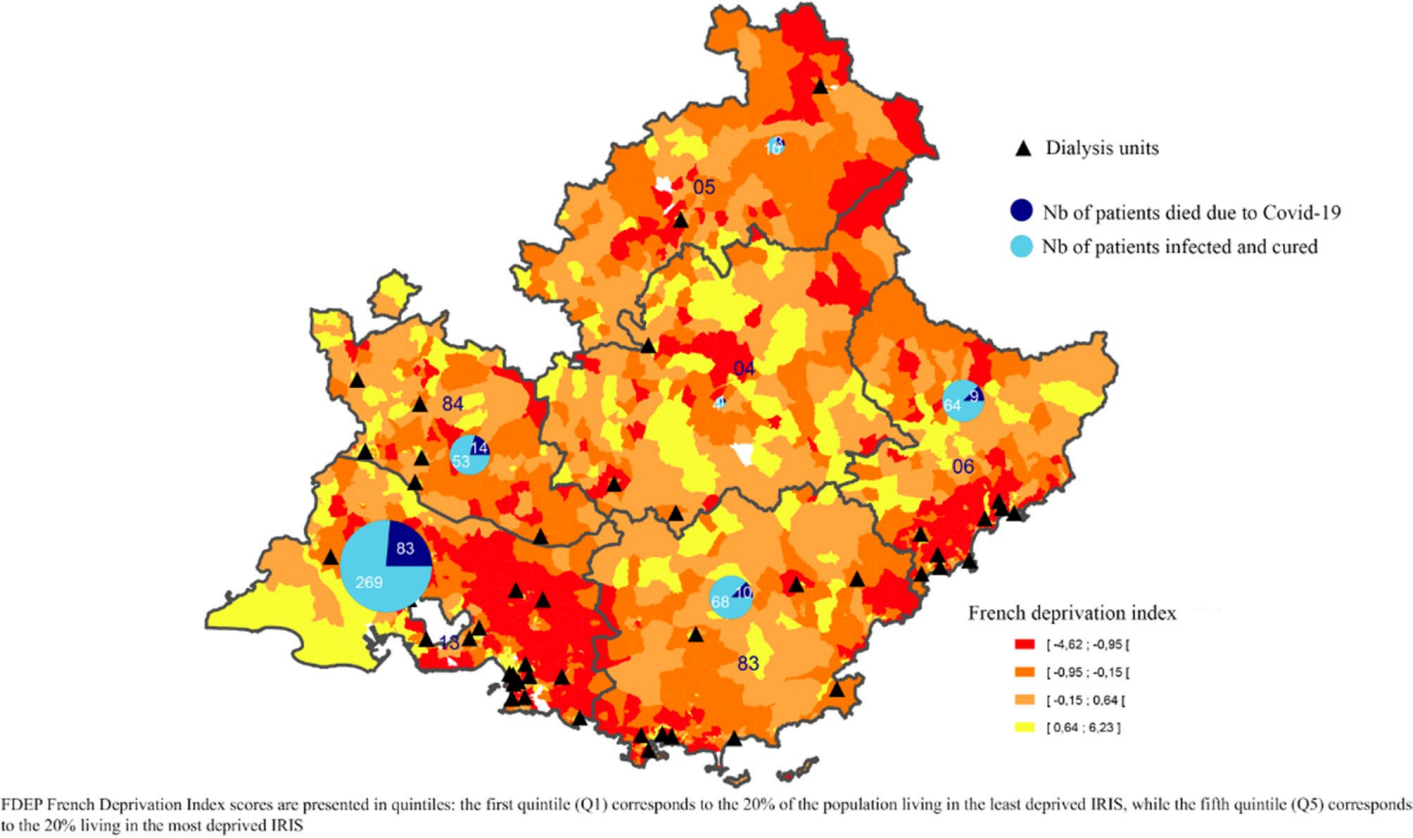
Distribution of the FDep, proportion of dialysis patients with COVID-19, lethality rate in PACA region

**Fig. 2:**
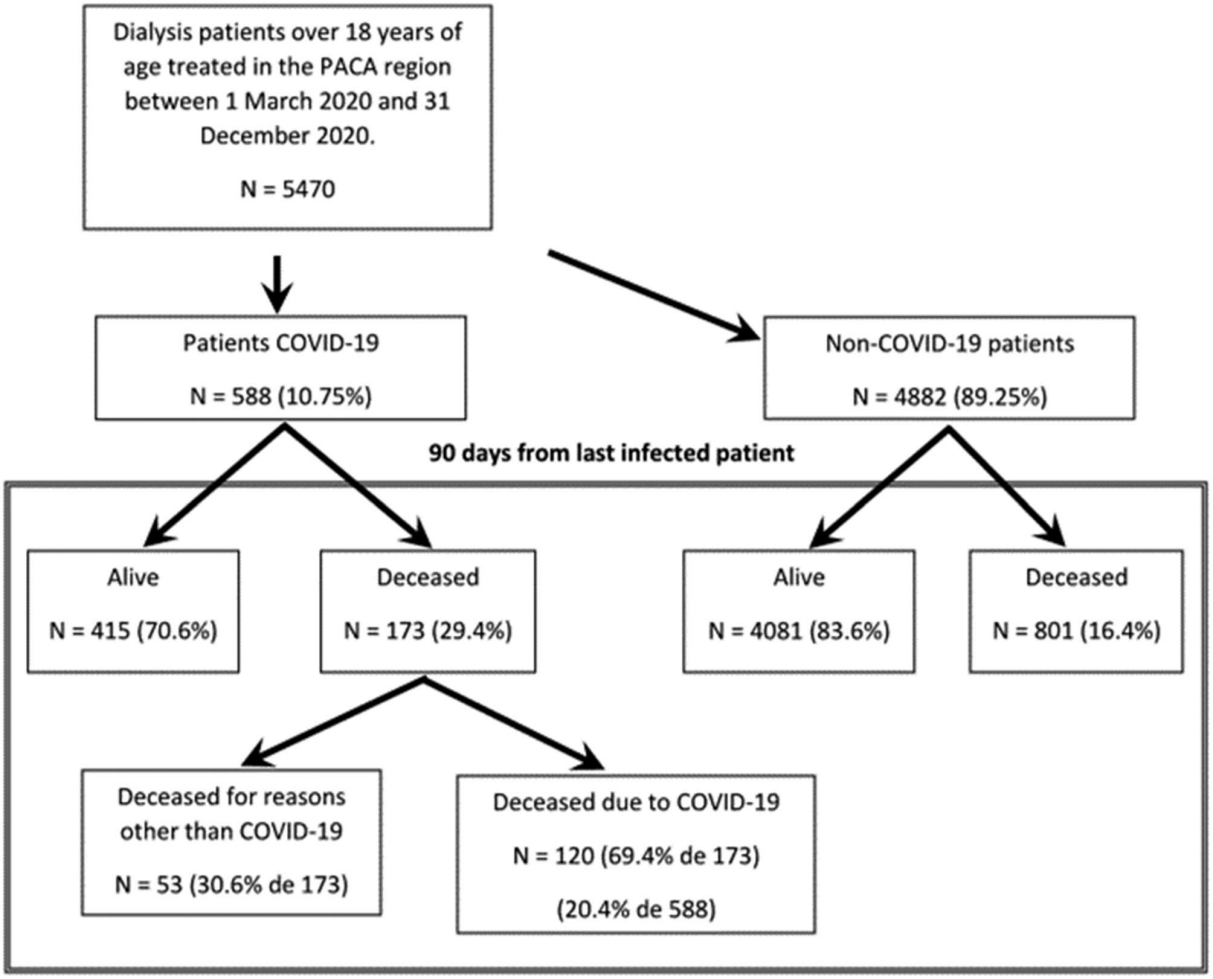
Evolution of the dialysis patient cohort

The PACA region (Fig. 1) is divided into different *departments* (an administrative area smaller than a region but larger than a commune/municipality) as follows: Alpes de Haute Provence, Hautes Alpes, Alpes Maritimes, Bouches du Rhône, Var and Vaucluse. Throughout the PACA region, 79 dialysis units were used to treat 5470 dialysis patients with ESRD during the study period (Fig. 2).

Data were extracted from the REIN registry [17, 21], together with additional data on dialysis patients infected with COVID-19. [19].

For our analysis, dialysis patients were divided into two groups: those infected with COVID-19 during the study period and those who were not.

### Description of covariates

#### Data extracted from the REIN registry for all dialysis patients.

For each dialysis patient, the following data were extracted from the REIN registry: age, sex, residential address, and clinical characteristics at the last follow-up, specifically body mass index, smoker status (never smoked, ex-smoker, current smoker), walking status (walked without assistance, required some assistance to walk, or was totally dependent on assistance to walk), registered on the kidney transplant waiting list, type of dialysis (haemodialysis and peritoneal dialysis), treatment modality (in-centre/hospital, in a satellite medical unit, in a self-care unit, at home and training), and number of years since first dialysis up to 31 December 2020 or until date of death.

The following comorbidities were also recorded: diabetes, cardiovascular disease (i.e., coronary insufficiency, myocardial infarction, heart rhythm or conduction disorders, heart failure), vascular disease (abdominal aortic aneurysm and/or stroke, transient ischaemic attack, lower limb arteritis), respiratory disease (i.e., chronic respiratory insufficiency and/or COPD, and/or sleep apnoea syndrome, and/or oxygen therapy and/or home respiratory assistance), progressive cancer, and haemopathy.

In order to determine the impact of the COVID-19 pandemic on mortality, all deaths of infected patients in the sample up to 90 days after infection notification were considered (i.e., up to 31 March 2021 for dialysis patients infected up to 31 in December 2020) [22].

For each COVID-19 infected patient, nephrologists recorded the date of diagnosis (indicating how the diagnosis was made), clinical status and treatment in the REIN registry. Clinical status and treatment were jointly coded: asymptomatic with no treatment, mild illness treated at home, moderate illness treated in hospital, severe illness treated in an intensive care unit (ICU), death. All changes in a patient’s clinical status (e.g., from mild to moderate illness, moderate to severe, etc.) were also recorded. The classification of COVID-19 clinical severity was based on a standardized definition disseminated by the Agence de la Biomédecine to all dialysis centers and used within the national REIN COVID-19 surveillance system This classification was derived from the World Health Organization COVID-19 Therapeutic Trial Synopsis, ensuring consistency with internationally recognized severity criteria. [23, 24]. This classification has been applied consistently across centers and has been used in previous REIN-based publications. For the data analysis, only the most severe clinical condition was selected for each patient. COVID-19-infected patients were then divided into two groups according to whether they were hospitalised or not.

#### Ecological indicators

The REIN registry does not collect data on individual social deprivation; accordingly, the latter was assessed using the French Deprivation Index (FDep) for each patient’s area of residence [25]. We describe this process in more detail below.

The FDep has four components: the percentage of employees in the labour force, the percentage of inhabitants aged 15 and over with a high school diploma, the unemployment rate in the labour force, and the median household income. Data for the first three components were taken from the 2015 French Census conducted by the National Institute for Statistics and Economic Studies (INSEE), while data for the median household income came from the national tax authority [26]. The spatial scale used to calculate the FDep was the French census block level (IRIS), a sub-municipal division developed by INSEE. This is the smallest geographical unit in France for which demographic and socioeconomic information is available from the national census. Each patient’s home address was matched to an IRIS, using the following website: http://www.geoportail.gouv.fr/donnees/iris. The FDep values are presented in quintiles: the first quintile (Q1) corresponds to the 20% of the population living in the least deprived IRIS, while the fifth quintile (Q5) corresponds to the 20% living in the most deprived IRIS [27]. Population density was calculated separately by IRIS because of its reported influence in the literature on the spread of COVID-19 infection, and because it is not included in the FDep.

#### Ethical permissions obtained

All persons included in our study were extracted from the French REIN registry. The latter was approved by two ethics committees: the French Data Protection Authority (CNIL) (authorization number 903, 188), and the Advisory Committee on Information Processing for Research Authorization (CCTIRS) (authorization number 03.149). All subjects provided verbal informed consent to participate.

#### Statistical analysis

Chi-squared tests, Fisher’s exact tests, and Wilcoxon-Mann-Whitney tests were used where appropriate to make comparisons between COVID-19-infected and non-infected dialysis patients. Individual and ecological (‘IRIS level, see above’) factors associated with COVID-19 infection (i.e., study outcome) were analysed using multivariable multilevel logistic modelling. First, a multilevel model without any covariate (i.e., null/empty model) was performed to test the significance of the IRIS-level variance and to assess whether the multilevel approach was justified [28]. A multilevel model adjusted for individual covariates only (i.e., individual model) has been fitted and a third model adjusted for both individual and IRIS covariates (full model) was then fitted. All models were adjusted for age and gender. Other individual factors were selected using backward selection (*p* < 0.05). At each step, the contextual effect was estimated using the median odds ratio (MOR) [29], which is the median value of the odds-ratio between IRIS at high risk of covid-19 infection and those with a lower risk, by randomly drawing two IRIS from the sample (the MOR is always ≥ 1). The intra-class coefficient (ICC) [30] was calculated to gauge the proportion of the total variance in the outcome attributable to the IRIS level. The proportional change in variance (PCV) [31] was used to measure the change in IRIS-level variance between the null model and the individual-level model, and between the individual-level model and the full-model including IRIS-level covariates.

To assess the robustness of the main findings, a sensitivity analysis was performed by stratifying the analyses according to epidemic wave (Wave 1: March–June 2020; Wave 2: September–December 2020). Because the number of COVID-19 cases was limited during Wave 1, multilevel models did not converge. We therefore fitted separate standard multivariable logistic regression models for each wave, using the same set of individual and ecological covariates as in the main analysis.

All analyses were based on two-sided (i.e., tailed) p-values, with statistical significance defined as *p* ≤ 0.05. They were performed using SAS 9.4 statistical software (SAS Institute, Cary, NC).

## Results

A total of 5470 dialysis patients aged 18 years and older were on dialysis in the PACA region between 1 March 2020 and 31 December 2020. Of these, 588 were infected with COVID-19, representing a prevalence of 10.7%. Of the 173 patients infected with COVID-19 who died during the study period, 120 deaths (20.4%) were due to the disease (Fig. 2).

The proportion of infected patients ranged from 3.1% (Alpes de Haute Provence) to 15.4% (Bouches du Rhone) in the region’s six different *departments* (Table 1). The mortality rate associated with COVID-19 infection ranged from 12.3% to 23.6%. More densely populated areas [26] had higher numbers of infected patients (Table 2).

**Table 1:** COVID-19 infection in dialysis patients in the six administrative departments of the PACA region (*n* = 5,470)

			Department						Total
			Alpes de Haute Provence	Hautes Alpes	Alpes Maritimes	Bouches du Rhône	Var	Vaucluse	
**Active dialysis patients during the study period**		**n**	**160**	**137**	**979**	**2285**	**1205**	**704**	**5470**
Infected	Nb of patients	**n**	**5**	**13**	**73**	**352**	**78**	**67**	**588**
		**%**	**3.1%**	**9.5%**	**7.5%**	**15.4%**	**6.5%**	**9.5%**	**10.7%**
	Death during the study period	**n**	2	6	14	118	15	18	173
		**%**	40.0%	46.1%	19.1%	33.5%	19.2%	26.8%	29.4%
	Death attributed to COVID-19	**n**	1	3	9	83	10	14	120
		**%**	20.0%	23.1%	12.3%	23.6%	12.8%	20.9%	20.4%
Population [26]	Nb. of Residents	**n**	165 232	140 349	1 088178	2 044355	1 079043	560 425	5 077582
	Population Density	**Inhab/km**^**2**^	24	25	253	402	181	157	162
Number of dialysis units		**n**	4	5	13	31	16	10	79

% calculated in column

**Table 2:** Socioeconomic situation according to COVID-19 status (*n* = 5,340)

	COVID-19 infection				p*	Total (n = 5340)	
	No (n = 4772)		Yes (n = 568)				
	n	%	n	%		n	%
**Population density(hbts/km**^**2**^**)**							
Mean(sd) Q1-Median-Q3 Quartiles	5883(8126) 343–2938–7802		7406(8865) 680–4113–9960		** < 0.001**	6045(8220) 356–3039–8012	
< Q1	1196	25.1	110	19.4	**0.003**	1306	24.5
Q1-Median	1200	25.1	125	22.0	0.111	1325	24.8
Median-Q3	1196	25.1	155	27.3	0.261	1351	25.3
> Q3	1180	24.7	178	31.3	**0.001**	1358	25.4
**FDep**							
Mean(sd) Q1-Median-Q3 Quintiles	0.18(1.57) −0.87–0.04–0.88		0.42(1.85) −0.88–0.06–1.48		**0.016**	0.2(1.6) −0.87–0.03–0.92	
Very low	1177	24.7	142	25.0	0.877	1319	24.7
Low	1122	23.5	113	19.9	0.058	1235	23.1
Medium	927	19.4	87	15.3	**0.017**	1014	19.0
High	634	13.3	77	13.6	0.845	711	13.3
Very high	912	19.1	149	26.2	** < 0.001**	1061	19.9

*: chi-square test, Fisher’s exact test, or Wilcoxon-Mann-Whitney test where appropriateFrench Deprivation Index (FDep)

Of the 5470 study patients, 130 had incomplete home addresses; accordingly, these persons could not be assigned to an IRIS and were therefore excluded from all statistical analyses including contextual variables. Of these 130 patients, 20 (15.4%) were infected with COVID-19; 11 died before 31 December 2020, and 8 of the 11 deaths were attributed to the disease.

The majority (95.8%) of infected dialysis patients were diagnosed by PCR; the remainder were diagnosed from clinical and radiological signs. Table 3 compares the socio-demographic and medical characteristics of COVID-19 and non-COVID-19 patients.

**Table 3:** Clinical characteristics of dialysis patients according to COVID-19 infection status (*n* = 5,470)

	COVID-19 infection				p*	Total (n = 5470)		
	No (n = 4882)		Yes (n = 588)					
	n	%	n	%		n		%
**Male**	3093	63.4	382	65.0	0.468	3475		63.5
**Age (years)**								
Mean(sd) Q1-Median-Q3	71.3(14.3) 63.5–73.8–82.2		72.1(14.0) 65.2–73.8–82.2		0.279	71.4(14.2) 63.7–73.8–82.2		
**Nb of years since first dialysis treatment on 31 December 2020 or at date of death**								
Mean(sd) Q1-Median-Q3	5.8(6.9) 1.4–3.4–6.9		5.2(6.8) 1.2–2.9–5.9		**0.016**	5.7(6.9) 1.4–3.3–6.8		
**BMI (kg/m**^**2**^**)**								
Mean(sd) Q1-Median-Q3	26.2(5.5) 22.3–25.4–29.3		26.6(5.6) 22.7–26.0–30.0		**0.048**	26.2(5.5) 22.3–25.4–29.4		
**BMI > 30 kg/m**^**2**^	1015	20.8	138	23.5	0.105	1153		21.1
**Smoking status**								
Missing/not specified	661	13.5	54	9.2		715		13.1
Never smoked	2302	47.2	319	54.3	**0.024**	2621		47.9
Smoker	649	13.3	52	8.8	** < 0.001**	701		12.8
Ex-smoker	1270	26.0	163	27.7	0.841	1433		26.2
**Diabetes**								
Missing/not specified	11	0.2	3	0.5		14		0.3
Yes	2103	43.1	309	52.6	** < 0.001**	2412		44.1
**Chronic respiratory disease**								
Missing/not specified	34	0.7	3	0.5		37		0.7
Yes	988	20.2	122	20.7	0.786	1110		20.3
**At least one cardiovascular disease**								
Missing/not specified	27	0.6	3	0.5		30		0.5
Yes	2571	52.7	336	57.1	**0.044**	2907		53.1
**At least one vascular disease**								
Missing/not specified	30	0.6	3	0.5		33		0.6
Yes	910	18.6	104	17.7	0.613	1014		18.5
**Progressive cancer or hemopathy**								
Missing/not specified	75	1.5	3	0.5		78		1.4
Yes	535	11.0	78	13.3	0.113	613		11.2
**On or more physical disability**								
Missing/not specified	140	2.9	13	2.2		153		2.8
Yes	1063	21.8	170	28.9	** < 0.001**	1233		22.5
**Walking capability status**								
Missing/not specified	219	4.5	18	3.1		237		4.3
Could walk without help	3649	74.7	394	67.0	< 0.001	4043		73.9
Required some assistance to walk	710	14.5	133	22.6	< 0.001	843		15.4
Dependent on assistance to walk	304	6.2	43	7.3	0.372	347		6.3
**Usual means of transport**								
Missing/not specified	86	1.8	6	1.0		92	1.7	
Ambulance	1315	26.9	216	36.7	< 0.001	1531	28.0	
LMV**, Taxi,	2990	61.2	329	56.0	0.007	3319	60.7	
Private car, public transport	491	10.1	37	6.3	0.002	528	9.7	
**On kidney transplant waiting list**								
Missing/not specified	24	0.5	2	0.3		26	0.5	
No	4217	86.4	541	92.0		4758	87.0	
Yes	641	13.1	45	7.7	** < 0.001**	686	12.5	
**Modality of dialysis location**								
Self-care unit	371	7.6	42	7.1	0.741	413	7.6	
Hospital center	2991	61.3	409	69.6	< 0.001	3400	62.2	
At Home	283	5.8	14	2.4	< 0.001	297	5.4	
Training	47	1.0	0	0.0	0.008	47	0.9	
Medical satellite unit	1190	24.4	123	20.9	0.066	1313	24.0	
**Dialysis vascular approach used**								
Missing/not specified	273	5.6	14	2.4		287	5.2	
Other	66	1.4	4	0.7	0.180	70	1.3	
Catheter tunnelled	1142	23.4	128	21.8	0.199	1270	23.2	
Native arteriovenous fistula	3172	65.0	409	69.6	0.250	3581	65.5	
Bypass	229	4.7	33	5.6	0.419	262	4.8	
**Method of dialysis**								
Peritoneal dialysis	268	5.5	14	2.4		282	5.2	
Haemodialysis	4614	94.5	574	97.6	0.001	5188	94.8	
**Deceased on 31 December 2020**								
No	4081	83.6	415	70.6		4496	82.2	
Yes	801	16.4	173	29.4	< 0.001	974	17.8	
**Died within 90 days of COVID-19 diagnosis diagnosis**								
No	NA		468	79.6				
Yes			120	20.4				

*: chi-square test, Fisher’s exact test, or Wilcoxon-Mann-Whitney test where appropriate. **: Light Medical Vehicle

There were no statistically significant differences in age or gender between both groups. On average, COVID-19 patients had been on dialysis for less time (5.2 vs 5.8 years, *p* = 0.016). Moreover, they were less likely to smoke (8.8% vs 13.3%, *p* < 0.001). In contrast, they were more likely to have a disability, diabetes, a higher BMI, and cardiovascular disease. They were less likely to be on the kidney transplant waiting list (7.7% vs 13.1%, *p* < 0.001) but more likely to be treated in a centre (69.6% vs 61.3%, *p* < 0.001) and to receive haemodialysis (97.6% vs 94.5%, *p* = 0.001). Furthermore, the mortality rate during the study period was significantly higher in COVID-19-infected patients on dialysis (29.4% vs 16.4%, *p* < 0.001).

In the subset of 5340 dialysis patients for whom ecological variables were available (Table 2), those infected with COVID-19 were more likely to live in an area with a high population density (31.3% vs 24.7%, *p* = 0.001) and a very high FDep (26.2% vs 19.1%, *p* < 0.001).

The null multilevel model (Table 4) showed an inter-IRIS variance that was significantly different from zero (σ = 0.27, *p* = 0.007), justifying the hierarchical approach used (see above). The inter-IRIS variance increased to 0.29 (+6.6%) after the introduction of the individual variables and remained significantly different from zero (*p* = 0.006). This result suggests that the individual covariates introduced in the individual model do not explain the inter-IRIS variance observed in the null model. The results of the individual model closely mirrored those of the univariate analysis. Specifically, COVID-19-infected patients had been on dialysis treatment for a shorter length of time (5.2 years *versus* 5.8 for the non-infected group) (odds ratio [OR] 95% confidence interval [CI]: 0.79 0.65–0.97), less likely to smoke (OR 95% CI: 0.64 0.47–0.88), and less likely to be on the transplant waiting list (OR 95% CI: 0.57 0.41–0.80). Conversely, they were more likely to be on haemodialysis (OR 95% CI: 2.29 1.31–3.99), to have diabetes (OR 95% CI: 1.33 1.10–1.61), and to have one or more physical disabilities (OR 95% CI: 1.35 1.10–1.67). In the full model, the inter-IRIS variance decreased to 0.23 (−18.5%) but remained significantly different from zero (*p* = 0.019). The MOR was estimated at 1.58, indicating that the residual variation in the odds of being infected with COVID-19 between two different IRIS increased by a factor of 1.6 when two dialysis patients with the same individual and contextual characteristics were randomly selected from two different IRIS. Compared with the individual model, the associations with individual covariates remained consistent. In terms of ecological factors, patients residing in IRIS with a high population density had higher odds of COVID-19 infection (OR 95% CI: 1.47–1.10–1.95), all other things being equal. Similarly, dialysis patients living in IRIS with a very high FDep had higher odds of COVID-19 infection (OR 95% CI 1.48 1.08–2.04).

**Table 4:** Individual and ecological factors associated with COVID-19 infection – multivariable multilevel logistic models (*n* = 5,340)

	Variable	Null- model	Individual- model		Full-model	
			OR95% CI*	p	OR95% CI*	p
**Individual** **factors**	Female (ref. Male)		0.93 0.77–1.13	0.471	0.92 0.76–1.11	0.379
	Age 60–69 (ref. < 60)		1.00 0.74–1.35	0.998	1.03 0.76–1.39	0.856
	Age 70–79 (ref. < 60)		0.99 0.75–1.30	0.927	1.03 0.78–1.36	0.819
	Age > 79 (ref. < 60)		0.93 0.70–1.23	0.625	0.99 0.75–1.31	0.939
	No. of years since first dialysis > Q3 (ref. < Q3)		0.79 0.65–0.97	0.022	0.78 0.64–0.96	0.017
	Smoker (ref. Never-smoker/ex-smoker/missing)		0.64 0.47–0.88	0.006	0.64 0.46–0.87	0.005
	Diabetes (ref. No)		1.33 1.10–1.61	0.003	1.31 1.08–1.58	0.006
	One or more physical disabilities (ref. No)		1.35 1.10–1.67	0.005	1.34 1.09–1.65	0.006
	On kidney transplant waiting list (ref. No)		0.57 0.41–0.80	0.001	0.59 0.42–0.83	0.002
	Haemodialysis modality (ref. Other)		2.29 1.31–3.99	0.004	2.20 1.26–3.84	0.005
**Ecological** **factors**	Population density Q1-Median (ref. < Q1)				1.09 0.82–1.45	0.556
	Population density Median-Q3 (ref. < Q1)				1.30 0.98–1.72	0.069
	Population density > Q3 (ref. < Q1)				1.47 1.10–1.95	**0.009**
	FDep quintiles “Very low” (ref. “Medium”)				1.32 0.97–1.79	0.073
	FDep quintiles “Low” (ref. “Medium”)				1.09 0.79–1.48	0.607
	FDep quintiles “High” (ref. “Medium”)				1.25 0.88–1.76	0.210
	FDep quintiles “Very high” (ref. “Medium”)				1.48 1.08–2.04	**0.014**
**Inter-IRIS** **variance**	Estimation (standard error)	0.27(0.12)	0.29(0.13)		0.23(0.13)	
	p-value	0.007	0.006		0.019	
	Median Odds Ratio (MOR)**	1.64	1.66		1.58	
	Intra Class Coefficient (ICC)***	7.5%	8.0%		6.6%	
	Proportional Change in Variance (PCV)****		6.6%		−18.5%	

* Adjusted odds ratios with 95% confidence interval ** MOR converts the Iris-level variance into the odds ratio scale. *** ICC estimates the proportion of the total variance attributed to the IRIS level. **** PCV measures the change in IRIS-level variance between the empty/null model and the individual-level model, as well as between the individual-level model and the full-model

In the COVID-19-infected group, 141 (24%) were asymptomatic, 144 (24.5%) had mild illness and were treated at home, 252 (42.8%) had moderate illness requiring hospitalisation and finally, 48 (8.2%) had severe illness requiring admission to an ICU.

Table 5 shows the factors associated with COVID-19-infected dialysis patients who required hospitalisation and/or ICU admission. The results showed that infected patients requiring hospitalisation and/or ICU admission were more likely to be male, to have diabetes, and to live in precarious housing conditions. They were also more likely to die within 90 days of diagnosis

**Table 5:** Individual and ecological factors associated with severity of the clinical status of COVID-19-infected dialysis patients

	Dialysis **patients hospitalised and/or in intensive care**			
	No (n = 285)		Yes (n = 300)	
	n	%	n	%
**Male**	166	58,2	**214**	71.3***
**Age (years)**				
Mean(sd)	71.1(15.1)		73.1(12.9)	
Q1-Median-Q3	64.4–72.7–81.9		66.0–74.7–82.2	
**Nb of years since first dialysis on 31 December 2020 or at date of death**				
Mean(sd)	5.5(7.1)		4.8(6.4)	
Q1-Median-Q3	1.3- 3.1- 6.4		1.2- 2.7–5.4	
**BMI kg/m**^**2**^				
Mean(sd)	26.3(5.7)		27.0(5.4)	
Q1-Median-Q3	22.2–25.6–29.7		23.1–26.4–30.4	
**BMI > 30 kg/m**^**2**^	65	22,8	73	24,3
**Smoking**				
*Missing/not specified*	32	11,2	21	7
Never smoked	151	53	166	55,3
Smoker	29	10,2	23	7,7
Ex-smoker	73	25,6	90	30
**Diabetes**	134	47	**175**	58.3***
**Chronic respiratory disease**	54	18,9	68	22,7
**At least one cardiovascular disease**	156	54,7	177	59
**At least one vascular disease**	53	18,6	49	16,3
**Progressive cancer or hemopathy**	37	13	41	13,7
**One or more physical disability**	86	30,2	83	27,7
**Walking capability status**				
Missing/not specified	7	2,5	11	3,7
Could walk without help	198	69,5	193	64,3
Required some assistance to walk	58	20,4	75	25
Dependent on assistance to walk	22	7,7	21	7
**Usual means of transport**				
Missing/not specified	5	1,8	1	0,3
Ambulance	93	32,6	121	40,3
LMV, Taxi,	170	59,6	159	53,0
Private car, public transport	17	6,0	19	6,3
**On kidney transplant waiting list**	26	9,1	19	6,3
**Modality of dialysis**				
Self-care unit	22	7,7	20	6,7
Hospital center	200	70,2	208	69,3
At Home	4	1,4	9	3,0
Medical satellite unit	59	20,7	63	21,0
**Vascular approach used for dialysis**				
Missing/not specified	5	1,8	8	2,7
Other	3	1,1	1	0,3
Catheter tunnelled	57	20,0	71	23,7
Native arteriovenous fistula	204	71,6	203	67,7
Bypass	16	5,6	17	5,7
**Method of dialysis: Hemodialysis**	280	98,2	292	97,3
**Died within 90 days of COVID-19 diagnosis**	22	7,7	**95**	31.7***
**Population density(hbts/km**^**2**^**)**				
Mean(sd)	7377(9040)		7123(8560)	
Q1-Median-Q3	507–3893– 10,238		1066–4007–9133	
Quartiles				
< Q1	58	20,4	55	18,3
Q1-Median	67	23,5	69	23
Median-Q3	72	25,3	85	28,3
> Q3	88	30,9	91	30,3
**FDep**				
Mean(sd)	0.2(1.8)		**0.5(1.9)**	
Q1-Median-Q3	−1.1–0.1- 1.0		-0.8–0.2–1.5*	
Quintiles				
Very low	81	28,4	71	23,7
Low	60	21,1	55	18,3
Medium	41	14,4	50	16,7
High	38	13,3	40	13,3
Very high	65	22,8	84	28

*p < 0.05, **p < 0.01, ***p < 0.001. Chi-square test, Fisher’s exact test, or Wilcoxon-Mann-Whitney test where appropriate (versus remaining of the sample) French Deprivation Index (FDep)

The results of the sensitivity analysis stratified by epidemic wave are presented in Supplementary File 1. Overall, the direction and magnitude of the associations observed in both Wave 1 and Wave 2 were broadly consistent with those of the main multilevel model. In particular, the associations between socioeconomic deprivation, population density, and the risk of COVID-19 infection remained stable across waves, supporting the robustness of the main findings.

## Discussion

This study confirms that socioeconomic deprivation significantly influenced both the transmission and severity of COVID-19 among dialysis patients in France, despite the fact that this population benefits from a regular and highly structured dialysis care pathway. More specifically, we found that patients living in the most socioeconomically deprived areas were more likely to be infected (OR 95% CI: 1.48, 1.08–2.04), and that unstable housing conditions were associated with a greater likelihood of hospitalization and/or admission to intensive care. Population density was also identified as a risk factor for both infection and severe disease (OR 95% CI: 1.47, 1.10–1.95).

These findings are particularly striking because dialysis patients, unlike many other populations studied during the pandemic, benefit from a continuous and highly structured care pathway once enrolled in dialysis. They attend dialysis units three times per week, receive systematic monitoring, and their care is fully reimbursed by the French national health insurance. This context substantially reduces, though does not entirely eliminate, the role of healthcare access as a driver of inequality, challenging the usual hypothesis that social inequalities act primarily through barriers to care access.

The persistence of a strong social gradient in infection and severity therefore suggests that other mechanisms are at play—notably housing conditions, occupational exposure, use of public transportation, and comorbidities associated with deprivation. As previous literature has shown [15, 32–36], people living in poverty are more likely to face environmental and behavioral risk factors that increase exposure and worsen prognosis, even when healthcare is accessible.

Our prevalence and lethality rates (10.7% and 20.4%, respectively) are consistent with national data and international studies in dialysis populations [37, 38]. Similarly, the associations we observed with male sex, diabetes, and vascular disease mirror existing findings, supporting the internal validity of our results.

This study has several limitations. First, due to the lack of individual-level socioeconomic data, we relied on an ecological indicator—the FDep index—which, although validated and widely used [25, 27], may introduce classification bias. In particular, area-level deprivation may not accurately reflect individual socioeconomic conditions (such as income, education, or employment status), potentially leading to ecological misclassification.

Second, asymptomatic infections may have gone undetected despite systematic testing, potentially underestimating true prevalence and overestimating lethality. Third, the small number of severe cases in our sample limited our ability to construct multivariate models for severity outcomes. Lastly, the analysis was confined to a single French region (PACA), which may limit generalizability. Nevertheless, the alignment of our findings with national data and the literature supports their robustness. The exclusion of patients with incomplete residential addresses, who could not be assigned to an IRIS, may have introduced a selection bias. These patients may disproportionately belong to socially vulnerable groups, potentially leading to an underestimation of the observed social gradients in COVID-19 infection and severity.

This study demonstrates that socioeconomic deprivation remained a significant determinant of both COVID-19 infection and clinical severity, even among patients benefiting from a highly structured and continuous dialysis care pathway. These findings challenge the assumption that healthcare access or continuity of care alone is sufficient to mitigate the impact of social inequalities on epidemic outcomes. The consistency of associations across epidemic waves further supports the robustness of these results.

Beyond documenting social inequalities in COVID-19 outcomes, which have been widely reported in the general population, this study addresses a less explored question: whether such inequalities persist within a care setting often considered relatively “protective.” By focusing on dialysis patients during the pre-vaccination period, our analysis helps disentangle the role of structural social determinants—such as living conditions, population density, and deprivation-related vulnerabilities—from healthcare access itself.

From a public health perspective, these findings remain highly relevant in 2025/2026. They suggest that preparedness for future epidemics cannot rely solely on ensuring access to or continuity of healthcare for chronically ill populations. Instead, epidemic response strategies should explicitly integrate social deprivation indicators into routine chronic disease management and crisis planning, in order to better identify high-risk groups and tailor preventive interventions. Although conducted in a single French region, this study offers insights applicable to other healthcare systems where access to care may also be considered universal, underscoring that effective response to health crises must address both medical and social determinants of health.

## Electronic supplementary material

Below is the link to the electronic supplementary material.

Supplementary Material 1

## Data Availability

Data could be provided to researchers by the corresponding author.

## Abbreviation

REIN: Renal Epidemiology and Information Network
FDep: French Deprivation index
PACA: Provence-Alpes-Côte d’Azur
COVID-19: coronavirus disease 2019
CKD: Chronic Kidney Disease
ESRD: End-Stage Renal Disease
INSEE: National Institute for Statistics and Economic Studies
CCTIRS: Advisory Committee on Information Processing for Research Authorization
MOR: Median Odds Ratio
ICC: Intra-Class Coefficient
PCV: Proportional Change in Variance
OD: Odds Ratio
CI: Confidence Interval
