# Supplementary material for: Beyond healthcare access: social deprivation and COVID-19 outcomes in dialysis patients in the provence-alpes-côte d’Azur region, France
Source: BMC Infect Dis. 2026 Feb 10;26:558. doi: 10.1186/s12879-026-12774-0 (PMC12990641; doi:10.1186/s12879-026-12774-0)
Supplement: Supplementary file 1 — Supplementary Material 1 [file 12879_2026_12774_MOESM1_ESM.docx]

| **Supplementary material. Individual and ecological factors associated with COVID-19 infection – Stratified Multivariable Logistic Models^†^** | | | | | |  |
| --- | --- | --- | --- | --- | --- | --- |
|  |  |  |  |  |  |  |
|  | **Variable** | **Wave 1  March-June 2020  (Wuhan-Hu-1 variant)**  **n=4,870** | | **Wave 2 September- December 2020**^‡^ **(B.1.160 variant)**  **n=5,222** | |  |
|  |  |  |  |  |  |  |
|  |  | **OR95% CI*** | **p** | **OR95% CI*** | **p** |  |
| **Individual** | Female (ref. Male) | 0.71 0.48;1.06 | 0.097 | 0.98 0.80;1.20 | 0.816 |  |
| **factors** | Age 60-69 (ref. <60) | 0.89 0.48;1.65 | 0.718 | 1.06 0.77;1.47 | 0.714 |  |
|  | Age 70-79 (ref. <60) | 0.93 0.53;1.62 | 0.789 | 1.04 0.77;1.41 | 0.778 |  |
|  | Age >79 (ref. <60) | 0.92 0.52;1.62 | 0.772 | 1.00 0.73;1.36 | 0.982 |  |
|  | No. of years since first dialysis >Q3 (ref. <Q3) | 1.19 0.81;1.76 | 0.367 | 0.70 0.56;0.87 | 0.002 |  |
|  | Smoker (ref. Never-smoker/ex-smoker/missing) | 0.32 0.14;0.74 | 0.008 | 0.74 0.53;1.04 | 0.080 |  |
|  | Diabetes (ref. No) | 1.24 0.84;1.83 | 0.279 | 1.32 1.07;1.62 | 0.009 |  |
|  | One or more physical disabilities (ref. No) | 1.11 0.72;1.73 | 0.627 | 1.40 1.12;1.76 | 0.003 |  |
|  | On kidney transplant waiting list (ref. No) | 0.70 0.36;1.34 | 0.284 | 0.57 0.39;0.84 | 0.004 |  |
|  | Haemodialysis modality (ref. Other) | 2.87 0.70;11.8 | 0.143 | 2.08 1.15;3.76 | 0.015 |  |
| **Ecological** | Population density Q1-Median (ref. <Q1) | 1.66 0.85;3.24 | 0.139 | 1.01 0.75;1.36 | 0.953 |  |
| **factors** | Population density Median-Q3 (ref. <Q1) | 2.09 1.10;3.98 | 0.025 | 1.16 0.87;1.55 | 0.312 |  |
|  | Population density >Q3 (ref. <Q1) | 2.94 1.56;5.56 | 0.001 | 1.25 0.93;1.68 | 0.141 |  |
|  | FDep quintiles “Very low” (ref. “Medium”) | 1.18 0.67;2.07 | 0.559 | 1.37 0.99;1.90 | 0.056 |  |
|  | FDep quintiles “Low” (ref. “Medium”) | 0.51 0.26;1.01 | 0.055 | 1.27 0.91;1.76 | 0.155 |  |
|  | FDep quintiles “High” (ref. “Medium”) | 0.76 0.38;1.53 | 0.440 | 1.39 0.97;2.00 | 0.074 |  |
|  | FDep quintiles “Very high” (ref. “Medium”) | 1.19 0.68;2.09 | 0.549 | 1.56 1.12;2.19 | 0.009 |  |
